# Association of mitochondrial respiratory chain enzymes with the risk and mortality of sepsis among Chinese children

**DOI:** 10.1186/s12879-021-07014-6

**Published:** 2022-01-06

**Authors:** Danni He, Ning Li, Xiuxiu Lu, Wei Li, Yuanmei Chen, Zhongyuan Sun, Lipeng Zhang, Linying Guo, Xiaodai Cui, Guowei Song, Wenquan Niu, Qi Zhang

**Affiliations:** 1 Institute of Clinical Medical Sciences, China-Japan Friendship Hospital, No. 2 Yinghua East Street, Chaoyang District, Beijing, 100029 China; 2 Department of Pediatrics, China-Japan Friendship Hospital, No.2 Yinghua East Street, Chaoyang District, Beijing, 100029 China; 3 Intensive Care Unit, Affiliated Children’s Hospital of Capital Institute of Pediatrics, Beijing, China; 4 Graduate School of Peking Union Medical College and Chinese Academy of Medical Science, Beijing, China; 5 Central Laboratory, Capital Institute of Pediatrics, Beijing, China

**Keywords:** Sepsis, Children, Mitochondrial Respiratory Chain Enzymes, Risk, Mortality

## Abstract

**Background:**

Sepsis is a leading cause of pediatric morbidity and mortality worldwide. The aim of this study was to explore the association of decreased mitochondrial respiratory chain enzyme activities with the risk for pediatric sepsis, and explore their association with mortality among affected children.

**Methods:**

A total of 50 incident cases with sepsis and 49 healthy controls participated in this study. The level of serum coenzyme Q10 was measured by high-performance liquid chromatography, and selected mitochondrial respiratory chain enzymes in WBC were measured using spectrophotometric. Logistic regression models were used to estimate odds ratio (OR) and 95% confidence interval (CI).

**Results:**

The levels of CoQ10, complex II, complex I + III and FoF1-ATPase were significantly higher in healthy controls than in children with sepsis (*p* < 0.001, = 0.004, < 0.001 and < 0.001, respectively). In children with sepsis, levels of CoQ10 and complex I + III were significantly higher in survived cases than in deceased cases (*p* < 0.001). Per 0.05 μmol/L, 50 nmol/min.mg and 100 nmol/min.mg increment in CoQ10, complex I + III and FoF1-ATPase were associated with significantly lowered risk of having sepsis, even after adjusting for confounding factors (OR = 0.85, 0.68 and 0.04, *p* = 0.001, < 0.001 and < 0.001, respectively). Per 0.05 μmol/L and 50 nmol/min.mg increment in CoQ10 and complex I + III was associated with significantly lowered risk of dying from sepsis during hospitalization, and significance retained after adjustment (OR = 0.73 and 0.76, 95% CI: 0.59 to 0.90 and 0.64 to 0.89, *p* = 0.004 and 0.001, respectively) in children with sepsis.

**Conclusions:**

Our findings indicate the promising predictive contribution of low serum CoQ10 and complex I + III to the risk of pediatric sepsis and its associated mortality during hospitalization among Chinese children.

*Trial registration* The trial was registered with www.chictr.org.cn, number ChiCTR-IOR-15006446 on May 05, 2015. Retrospectively registered.

**Supplementary Information:**

The online version contains supplementary material available at 10.1186/s12879-021-07014-6.

## Background

Sepsis is a leading cause of morbidity and mortality in children worldwide, with the case-fatality rate of 31.7% in developing countries and 19.3% in developed countries [1, 2]. In China, the incidence rate of pediatric sepsis was estimated to be 181/100,000 in 2014 [3]. The resolution of the World Health Assembly in 2017 has stressed the importance of developing more tools for sepsis diagnosis and treatment [4]. Current diagnostic strategies for identifying patients with sepsis mainly rely on clinical manifestations and markers of end-organ dysfunction, and the latest research has found that heat-shock-protein (HSP) 90α and human glucocorticoid receptor are also closely related to the death rate or severity of illness [5–7]. Since the definition, guidelines, biomarkers and pathways are constantly being improved and updated, early detection of sepsis using powerful tools or sensitive biomarkers and individualized close monitoring of patients at risk are of clinical and public health importance [8, 9].

Mitochondrial dysfunction is a feature of many pathologies, including sepsis [10–12]. Recent studies have shown a sharp decline in mitochondrial respiration of peripheral blood mononuclear cells in children with sepsis [13]. Moreover, mitochondrial dysfunction is also associated with kidney and liver injuries in patients with sepsis [14–16]. Besides, animal studies have shown that the reduction of mitochondrial calcium uptake was associated with the survival rate of rats [17]. Currently, most mitochondrion-sepsis correlation studies have focused on mitochondrial respiration or mitochondrial DNA [18, 19], yet few studies examined the enzymes and complexes on respiratory chains.

To fill this gap in knowledge and yield more information for future studies, we enrolled children suffering sepsis and healthy controls and assayed the activities of major mitochondrial respiratory chain enzymes in circulation, aiming to explore the association of mitochondrial respiratory chain enzyme activities with the risk for pediatric sepsis, and explore their association with hospitalized mortality among affected children.

## Methods

### Study Subjects

All study subjects were recruited from the Children’s Hospital Affiliated to the Capital Institute of Pediatrics during the period from March 2013 to August 2014. This study received approval from the Ethics Committee of this hospital, and was conducted in compliance with the Declaration of Helsinki. This study complied with the STROBE standard as was listed in Additional file 1: eFile S1.

A total of 50 children who were clinically confirmed to have sepsis were classified as the case group, and 49 age- and sex-matched healthy controls who had no signs of sepsis formed the control group. Healthy controls were from those undergoing health check-ups at the health clinic in our hospital. All study subjects in control group had no physical discomfort and abnormal indexes in blood routine or biochemical examinations.

### Tissue collection and diagnosis

**Fig. 1:**
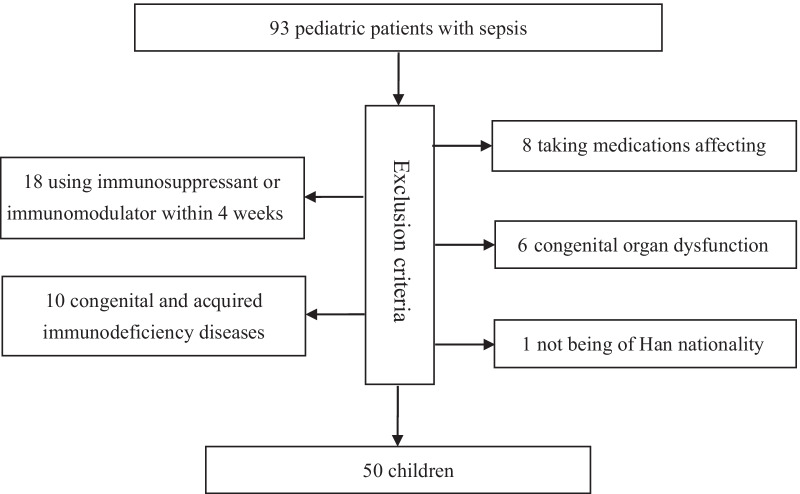
Flow diagram of included participants

Children aged 1 month to 192 months who were admitted to the Pediatric Intensive Care Unit (PICU) diagnosed with sepsis (Sepsis was SIRS in the presence of or as a result of suspected or proven infection. Severe Sepsis was defined as the presence of two or more systemic inflammatory response syndrome criteria, documented or suspected infection, and evidence of sepsis-induced organ dysfunction or tissue hypoperfusion as defined by International pediatric sepsis consensus conference: Definitions for sepsis and organ dysfunction in pediatrics [20]) were consecutively enrolled. For the definition of sepsis, please see Additional file 2: eFile S2. Children were excluded due to the following reasons: (i) taking medications affecting mitochondrial function, such as adenosine, ATP, CoQ10 inhibitors or modeling inhibitors; (ii) using immunosuppressant or immunomodulator within 4 weeks; (iii) congenital organ dysfunction; (iv) congenital and acquired immunodeficiency diseases; (v) not being of Han nationality. The participant flow diagram was shown in Fig. 1.

### Baseline characteristics and clinical biomarkers

Baseline information was abstracted from the medical record system. Blood samples were collected from all affected children in the first 24 h of admission and stored with ethylenediaminetetraacetic acid (EDTA) at 4℃. Then blood samples were centrifuged at 2000 rpm for 10 min to separate the serum frozen at −80 ℃ within 4 h.

The following markers were assayed from blood samples: complete blood count, electrolytes, lymphocyte cells, IgM, IgA, IgG, C-Reactive Protein (CRP), Procalcitonin (PCT), serum coenzyme Q10 level (using high-performance liquid chromatography), Laboratories of BIOTECNOVO (Methanol (HPLC) Sigma-Aldrich America), and selected mitochondrial respiratory chain enzymes in WBC measured (using spectrophotometric method, Laboratories of BIOTECNOVO, Sigma-Aldrich America). Pediatric Critical Illness Score (PCIS) was tested at the time of the first diagnosis of sepsis, and vital status was determined at the time of discharge from PICU. Blood samples of healthy controls were collected on the date of physical examination. Serum coenzyme Q10 concentration levels and mitochondrial complex activities were assayed at the Institute of Experimental Science, and according to the signed informed consent form, children in the healthy group only extracted these indicators.

As mentioned earlier, it is used high-performance liquid Chromatography to measure CoQ10 levels [21]. In a nutshell, 25 µL of ethanol solution included 12.5 ng CoQ9 (used as internal standard) in an eppendorf tube was blended wit 25 µL of defrosted plasma. After adding 450 µL 1-Propanol, the tube vortexes were mixed for 2 min, then the high-speed was centrifuge in a cold room for 10 min. The divisible (~ 300 µL) superclear is filtered by a 0.2 µm filter column and transferred to an HPLC injection flask and a divisible 50 µL extract injected into an HPLC system equipped with a C18 reversed phase column and ESA Coulochem II electrochemical detector(ESA, Inc., Chelmsford, MA).In our study measured the plasma CoQ10 in 49 age- and sex-matched healthy controls,and discovered the mid-value of 0.88 µmol L − 1 (IQR 0.82–0.95),which is similar to the establishment of health control ranges in the literature (1.04 ± 0.33 µmol L − 1).

Once plasma was collected, peripheral blood mononuclear cells (PBMCs) were segregated by high speed centrifuge [22]. PBMCs were used to measure mitochondrial, apoptotic, and oxidative stress markers; fresh cells were used to measure mitochondrial membrane potential and oxygen consumption, and frozen cells were used for the remaining measures. All PBMCs were normalized to protein content measured according to the Bradford protein dye binding based method [23].The enzymatic mitochondrial complexes were measured by thermostat zed spectrophotometry at 37 ℃ according to the methodology of Rustin et al. [24], slightly modified for complex IV measurement in minute amounts of biological samples [25]. Measurement the cells requiring treatment prior to complex I and triton activities, digitalis saponin detergent was used to obtain specific mitochondrial NADH-dehydrogenase activity [26]. Mitochondrial respiratory chain enzyme was represented as nanomoles substrate or produce products that consume and milligramme of protein per minute.

### Statistical analyses

The χ^2^ tests for categorical data and Wilcoxon rank-sum tests for continuous data were used to assess the baseline characteristics among deceased and survived in patients with sepsis, as well as between survived and deceased children during hospitalization. Logistic regression analyses were conducted to assess the association of serum coenzyme Q10 and mitochondrial complex activities with the risk and mortality risk of sepsis at a significance level of 5% before and after adjusting for age and gender. Effect size estimates are expressed as odds ratio (OR) and 95% confidence interval (95% CI).

To test the performance of Logistic regression model, statistical indexes from calibration and discrimination aspects were adopted. Calibration capability was evaluated using the −2 log-likelihood ratio test, Akaike information criterion (AIC), and Bayesian information criterion (BIC) [27] to see how closely the prediction probability for the addition of serum coenzyme Q10 and mitochondrial complex activities reflected the actual observed risk and the global fit of modified risk model. Net reclassification improvement (NRI) and integrated discrimination improvement (IDI) [28, 29] were calculated to judge the discrimination capability of serum coenzyme Q10 and mitochondrial complex activities. Receiver operating characteristic (ROC) curves [30] were plotted based on the basic model and the plus of each enzyme detection index.

Finally, a prediction nomogram model was constructed based on factors of significance using the “rms” package in the open-source R software, version 3.5.1 (available at the website: https://www.r-project.org).

Unless otherwise indicated, STATA software Release 14.1 (Stata Corp, TX, USA) was used for statistical analyses. A P value of less than 0.05 was considered statistically significant, and for multiple comparisons, Bonferroni correction method was used.

## Results

### Baseline characteristics

This study included 50 children with sepsis with an average age of 27.21 months, including 36 (72.0%) males. The control group contained 49 gender, age-matched healthy children with an average age of 26.72 months, containing 35 (71.4%) males (P > 0.05). The baseline characteristics between deceased and survived children with sepsis are presented in Table 1.

**Table 1:** Baseline characteristics of study participants in this study

Characteristics	Deceased	Survived	P
Age, months	26 ± 37.13	27.32 ± 35.23	0.835
Male, n (%)	11 (68.8%)	25 (73.5%)	0.726
SOFA, n (%)			0.047
0 to 10	3 (18.8%)	19 (55.9%)	
10 to 20	11 (68.8%)	13 (38.2%)	
> 20	2 (12.5%)	2 (5.9%)	
PCIS, n (%)			0.005
80 to 100	2 (12.5%)	19 (55.9%)	
71 to 80	4 (25%)	8 (23.5%)	
0 to 70	10 (62.5%)	7 (20.6%)	
MODS > 4 Organs, n (%)	14 (87.5%)	14 (41.2%)	0.002
MOF > 3 Organs, n (%)	11 (68.8%)	9 (26.5%)	0.004
DIC, n (%)	14 (87.5%)	22 (64.7%)	0.094
Shock, n (%)	13 (81.3%)	23 (67.6%)	0.318
ARDS, n (%)	12 (75%)	19 (55.9%)	0.194
Brain Failure, n (%)	14 (87.5%)	21 (61.8%)	0.064
Respiratory Failure, n (%)	13 (81.3%)	26 (76.5%)	0.704
Heart Failure, n (%)	11 (68.8%)	8 (23.5%)	0.002
Liver Failure, n (%)	12 (75%)	6 (17.6%)	< 0.001
Kidney Failure, n (%)	6 (37.5%)	5 (14.7%)	0.07
Stomach Failure, n (%)	12 (75%)	14 (41.2%)	0.026
Pancreas Failure, n (%)	3 (18.8%)	1 (2.9%)	0.055
Metabolic Acidosis, n (%)	12 (75%)	16 (47.1%)	0.063
Th1 cell (n/μl)	4.2 (1.9 to 6.5)	2.3 (1.9 to 2.6)	0.308
Th2 cell (n/μl)	0.3 (0.1 to 0.6)	0.1 (0.1 to 0.2)	0.075
CD3 (n/μl)	60 (53 to 71)	60 (40 to 73)	0.721
CD4 (n/μl)	32.5 (22 to 44)	23 (17 to 41)	0.251
CD8 (n/μl)	21.5 (17 to 27)	23 (18 to 33)	0.818
CD4/CD8	1.55 (0.87 to 2.19)	1 (0.68 to 1.5)	0.284
CD19 (n/μl)	27.4 (24.4 to 41)	28 (9 to 46)	0.858
CD16/CD56	6.5 (3 to 13)	9 (3 to 22)	0.294
IgG (g/L)	8.86 (6.46 to 13.76)	14.47 (10.26 to 23.61)	0.039
IgA (g/L)	0.8 (0.31 to 1.28)	0.36 (0.19 to 0.81)	0.260
IgM (g/L)	0.84 (0.53 to 1.09)	0.53 (0.35 to 0.76)	0.061
TRIG (mmol/L)	1.51 (0.97 to 2.39)	2.22 (1.5 to 2.69)	0.105
HDL (mmol/L)	0.81 (0.59 to 1.03)	0.69 (0.41 to 0.96)	0.173
LDL (mmol/L)	2.21 (1.63 to 2.79)	3.1 (2.17 to 3.79)	0.038
ALT (U/L)	28 (17.45 to 68.55)	168.5 (37.3 to 181.1)	0.003
AST (U/L)	50.15 (30.95 to 121.9)	104.1 (65.45 to 188.2)	0.105
LD (U/L)	320 (260 to 828)	874 (660 to 1089)	0.016
HBDH (U/L)	293.5 (199 to 741)	539 (413 to 749)	0.118
CK (U/L)	44.5 (32 to 77.5)	59 (20 to 142)	0.836
CKMB (ng/mL)	18 (12 to 27)	23.5 (18 to 38)	0.075
Glucose (mmol/L)	5.61 (4.81 to 6.74)	5.6 (3.2 to 8.15)	0.639
Ca (mmol/L)	2.26 (2.08 to 2.35)	1.96 (1.81 to 2.28)	0.015
WBC (10^9/L)	12.9 (8.14 to 16.65)	11.8 (7.02 to 16.96)	0.920
CRP (mg/mL)	8 (2 to 54)	23 (15 to 34)	0.116
PCT (ng/mL)	1.38 (0.27 to 2.55)	1.7 (0.1 to 2.89)	0.958

P values are calculated by nonparametric Wilcoxon rank sum tests for continuous variables expressed as median (interquartile range) and χ^2^ tests for categorical variables expressed as count and percent

*SOFA* Sequential organ failure score, *PCIS* Pediatric critical illness score, *MODS* Multiple organ dysfunction syndrome, *MOF* Multiple organ failure, *DIC* Disseminated intravascular coagulation, *ARDS* Acute respiratory distress syndrome, *TRIG* Triglyceride, *HDL* High-density lipoprotein, *LDL* Low density lipoprotein, *ALT* Alanine transaminase, *AST* Aspartate aminotransferase, *LD* Lactate dehydrogenase, *HBDH* Hydroxybutyrate dehydrogenase, *CK* Creatine Kinase, *CKMB* Creatine phosphokinase-Mb, *WBC* White blood cell, *CRP* C-Reactive Protein, *PCT* Procalcitonin

Distributions and comparisons of the activities of mitochondrial respiratory chain enzymes between healthy controls and children with sepsis, as well as between deceased and survived children during hospitalization, are shown in Table 2. The levels of CoQ10, complex II, complex I + III and FoF1-ATPase were significantly higher in controls than in cases (P: < 0.001, 0.004, < 0.001 and < 0.001, respectively). In children with sepsis, levels of CoQ10 and complex I + III were significantly higher in survived cases than in deceased cases (both P < 0.001).

**Table 2:** The comparisons of circulating biomarkers between children with sepsis and controls, as well as between survived and deceased in children with sepsis

Biomarkers	Controls	Children with sepsis			**P**_**1**_	**P**_**2**_
		All	Deceased	Survived		
CoQ10 (μmol/L)	1.0 (0.8 to 1.2)	0.7 (0.5 to 1.0)	0.5 (0.4 to 0.6)	0.8 (0.6 to 1.1)	< 0.001	< 0.001
Complex I (nmol/min.mg)	107.7 (95.2 to 120.8)	107.1 (98.8 to 124.4)	113.8 (98.5 to 125.0)	106.2 (98.8 to 124.4)	0.460	0.371
Complex II (nmol/min.mg)	99.6 (88.0 to 108.1)	88.2 (78.8 to 100.7)	87.1 (70.1 to 98.5)	91.3 (79.9 to 101.4)	0.004	0.499
Complex II + III (nmol/min.mg)	186.3 (170.2 to 202.7)	184.9 (158.8 to 199.8)	186.8 (132.2 to 207.5)	182.3 (163.6 to 199.6)	0.260	0.606
Complex IV (nmol/min.mg)	136.3 (123.1 to 155.7)	143.6 (88.1 to 166.6)	149.2 (88.8 to 164.1)	132.3 (88.1 to 166.8)	0.521	0.983
Complex I + III (nmol/min.mg)	898.1 (806.9 to 1083.1)	622.8 (384.7 to 811.9)	236.8 (132.6 to 592.5)	731.7 (563.4 to 823.4)	< 0.001	< 0.001
FoF1-ATPase (nmol/min.mg)	751.9 (605 to 903.3)	356.6 (222.8 to 544.7)	383.9 (215.3 to 710.8)	335.2 (222.8 to 535.1)	< 0.001	0.486

P values are calculated by t-test for normal variables and nonparametric Wilcoxon rank sum tests for non-normal data expressed as median (interquartile range). P_1_: Children with sepsis versus controls; P_2_: Survived versus deceased

*CoQ10* Coenzyme Q10

### Sepsis risk and mortality risk

The association of mitochondrial respiratory chain enzymes with sepsis development and mortality is shown in Table 3 before and after confounding adjustment. Per 0.05 μmol/L, 50 nmol/min.mg and 100 nmol/min.mg increment in CoQ10, complex I + III and FoF1-ATPase (a rotary molecular motor driven by ATP-hydrolysis) were associated with significantly lowered risk of having sepsis, even after adjusting for age and sex (OR = 0.85, 0.68 and 0.53, P: 0.001, < 0.001 and < 0.001, respectively), yet the association of complex II and complex II + III with sepsis risk was only marginally significant.

**Table 3:** Risk prediction of circulating biomarkers for the risk of sepsis, as well as the mortality risk

Significant risk factors	cOR	95% CI	P	aOR	95% CI	P^*^
Children with sepsis versus controls						
CoQ10 (+ 0.05 μmol/L)	0.88	0.82 to 0.95	0.001	0.85	0.77 to 0.93	0.001
Complex I (+20 nmol/min.mg)	1.19	0.75 to 1.89	0.456	1.02	0.59 to 1.78	0.942
Complex II (+50 nmol/min.mg)	0.29	0.10 to 0.85	0.025	0.29	0.10 to 0.85	0.025
Complex II + III (+50 nmol/min.mg)	0.71	0.39 to 1.28	0.258	0.42	0.20 to 0.90	0.026
Complex IV (+ 100 nmol/min.mg)	0.69	0.23 to 2.10	0.517	0.25	0.06 to 1.04	0.057
Complex I + III (+ 50 nmol/min.mg)	0.71	0.61 to 0.83	< 0.001	0.68	0.57 to 0.82	< 0.001
FoF1-ATPase (+ 100 nmol/min.mg)	0.53	0.42 to 0.68	< 0.001	0.53	0.40 to 0.70	< 0.001
Children with sepsis: deceased versus survived						
CoQ10 (+ 0.05 μmol/L)	0.74	0.61 to 0.90	0.003	0.73	0.59 to 0.90	0.004
Complex I (+ 20 nmol/min.mg)	1.35	0.71 to 2.58	0.364	1.38	0.71 to 2.65	0.340
Complex II (+ 50 nmol/min.mg)	1.30	0.36 to 4.80	0.690	1.32	0.35 to 4.96	0.678
Complex I + III (+ 50 nmol/min.mg)	0.81	0.37 to 1.77	0.598	0.79	0.33 to 1.85	0.580
Complex IV (+ 100 nmol/min.mg)	1.15	0.29 to 4.59	0.841	1.14	0.27 to 4.80	0.857
Complex I + III (+50 nmol/min.mg)	0.79	0.68 to 0.91	0.001	0.76	0.64 to 0.89	0.001
FoF1-ATPase (+ 100 nmol/min.mg)	1.08	0.87 to 1.35	0.478	1.08	0.87 to 1.35	0.481

*cOR* crude odds ratio, *aOR* adjusted odds ratio, *95% CI* 95% confidence interval

*P was calculated after adjusting for age and sex. Data are expressed as odds ratio, 95% confidence interval, P value

In children with sepsis, per 0.05 μmol/L and 50 nmol/min.mg in CoQ10 and complex I + III was associated with significantly lowered risk of dying from sepsis during hospitalization, and significance retained after controlling for age and sex (OR = 0.73 and 0.76, 95% CI: 0.59 to 0.90 and 0.64 to 0.89, P = 0.004 and 0.001, respectively). No signs of significance were noted for the other biomarkers.

### Accuracy appraisal of mortality prediction

The prediction accuracy gained by adding mitochondrial respiratory chain enzymes individually to the basic model is summarized in Table 4. As reflected by both calibration and discrimination statistics, only CoQ10 and complex I + III exhibited significant contribution to the mortality risk of hospitalized children with sepsis. Prediction accuracy was reinforced after adding both biomarkers simultaneously to the basic model.

**Table 4:** Prediction accuracy for sepsis mortality risk gained by adding each circulating biomarker to basic model

Statistics	Basic Model	Basic Model Plus							
		CoQ10	Complex I	Complex II + III	Complex II + III	Complex IV	Complex I + III	FoF1-ATPase	CoQ10 and Complex I + III
Calibration									
AIC	56.67	44.57	50.70	49.36	49.04	50.49	43.06	49.84	39.96
BIC	68.14	57.95	64.08	62.74	62.43	63.87	56.44	63.23	55.25
LR	Ref	14.1	7.97	9.31	9.62	8.18	15.61	8.82	20.71
LR (P)	Ref	< 0.001	0.005	0.002	0.002	0.004	< 0.001	0.003	< 0.001
Discrimination									
NRI	Ref	0.032	0.317	0.083	0.157	0.498	0.046	0.632	0.005
IDI	Ref	0.003	0.808	0.265	0.575	0.392	0.006	0.377	< 0.001
ROC area	0.881	0.932	0.881	0.884	0.893	0.888	0.928	0.903	0.950
ROC curve P	Ref	0.143	0.389	0.864	0.593	0.731	0.185	0.613	0.047

*AIC* Akaike information criterion, *BIC* Bayesian information criterion, *LR* likelihood ratio, *NRI* net reclassification improvement, *IDI* integrated discrimination improvement, *ROC* Relative operating characteristic curve, *Ref.* reference group. Basic model included age, gender, Pediatric critical illness score (Day 1), number of multiple organ failure, and shock

### Nomogram prediction model

**Fig. 2:**
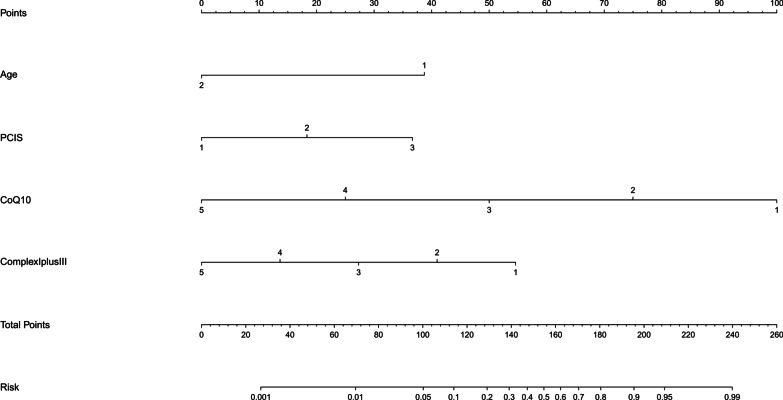
Nomogram prediction model of significant attributes for mortality risk in children with sepsis

In light of the significant contribution of low serum CoQ10 and complex I + III to mortality risk, a nomogram prediction model incorporating age, PCIS, CoQ10 and complex I + III was constructed (Fig. 2). The accuracy of this model reached as high as 92.3% (P < 0.001).

## Discussion

The aim of this study was to explore the association of mitochondrial respiratory chain enzyme activities with the risk of sepsis and its associated mortality risk among hospital-based children. The key findings are the promising predictive contribution of low serum CoQ10 and complex I + III levels to the risk of pediatric sepsis and its associated mortality during hospitalization, highlighting the importance of mitochondrial respiratory chain enzymes in the development and progression of pediatric sepsis. To the best of our knowledge, this is the first study that has evaluated the association of circulating mitochondrial respiratory chain enzyme activities with sepsis risk in the literature.

Currently, the biological implications of mitochondrial dysfunction in sepsis have been widely evaluated [10, 31, 32]. Mitochondria produce ATP by transferring electrons from substrates sequentially across four respiratory chain complexes (I to IV) and two mobile carriers (coenzyme Q and cytochrome C) to final electron acceptors [33]. Mitochondrial dysfunction is deemed as a key cellular event involved in the pathogenesis of multi-organ failure in sepsis, and it is secondary to tissue hypoxia and involves various toxins or mediators of inflammation that impair oxygen utilization (cytopathic hypoxia) [33, 34]. There is evidence that damaged mitochondria contribute to NACHT, LRR and PYD domains‑containing protein 3 (NLRP3) inflammasome‑related sepsis [31]. In addition, hydrogen was found to alleviate mitochondrial dysfunction and cytokine release via autophagy‑mediated NLRP3 inflammasome inactivation [13]. On the basis of above evidence, it is reasonable to hypothesize that mitochondrial dysfunction plays a contributory role in the development of sepsis, especially the abnormal expressions of mitochondrial respiratory chain complexes and mobile carriers. In support of this hypothesis, Donnino and colleagues conducted a randomized, double-blind, placebo-controlled, pilot trial, by showing that plasma CoQ10 levels were increased in patients with severe sepsis or septic shock, with the administration of oral ubiquinol [35]. However, the contribution of mitochondrial respiratory chain complexes and mobile carriers to the development and progression of sepsis has been rarely reported. To shed some light, we assayed the activities of mitochondrial respiratory chain enzymes in circulation among children with and without clinically-confirmed sepsis to examine their association with the risk and mortality of pediatric sepsis.

After a comprehensive analysis, we interestingly found that high CoQ10 and complex I + III levels were significantly associated with the reduced risk of having pediatric sepsis, as well as the reduced risk of dying from sepsis during hospitalization. CoQ10 has been proposed as an effective agent for reducing the deleterious effects of septic shock by acting as an oxygen free radical scavenger and thus stabilizing mitochondrial membranes, as well as by inhibiting the arachidonic acid metabolic pathway and the formation of various prostaglandins. There is evidence that CoQ10 is effective in alleviating histological organ damage in sepsis via mortality statistics of mice model [36]. In addition, animal studies indicated that complex I + III activity was higher in the sepsis groups than healthy controls in septic mice models caused by lipopolysaccharide, and after treatment with Simvastatin, mitochondrial complex I + III expression was increased [37]. Given the significant association observed in this present study and strong biological implications, it would be tempting to speculate that dysregulation of mitochondrial respiratory chain, in particular CoQ10 and complex I + III, is attributable to the pathogenesis of pediatric sepsis. Moreover, considering the high mortality rate of sepsis in children, identification of circulating biomarkers is of great importance in sanitary science and public health.

Although CoQ10 cannot be bedside-tested at present, it can be administered exogenously to improve the low-level of CoQ10 in septic children. Currently, oral CoQ 10 has been reported to inhibit the inflammatory response in migraine in a randomized double-blind placebo-controlled clinical trial [38]. Meanwhile, due to the important role of CoQ10 in the mitochondrial electron transport chain oxidation respiratory chain, it has shown useful effects in other interventional experiments such as polycystic ovary syndrome, fibromyalgia et al. [39, 40]. Based on the available evidence, it is reasonable to believe that CoQ10 opens the potential for future therapeutic interventions in septic children. In addition, to improve the serum CoQ10 by supplementing appropriate vitamins B2, B9, B12, and C are all possible to improve pediatric sepsis by maintaining mitochondrial function [41]. Additional research are worth designing to investigate the potential of CoQ10 as a therapeutic agent in children with sepsis.

Several limitations should be acknowledged for this study. First, the small sample size involved may limit the power to detect small contributions. Second, all study participants are of Chinese descent, which may limit the extrapolation of our findings to other ethnic groups. Third, only a death-or-discharge outcome was recorded during hospitalization and further follow-up evaluation was not available for us. At the same time, this research lack laboratory indicators for the healthy group. Fourth, mitochondrial respiratory chain enzyme activities were assayed only once, and their dynamic monitoring is of added interest.

Taken together, our findings indicate the promising predictive contribution of low serum CoQ10 and complex I+III to the risk of pediatric sepsis and its associated mortality during hospitalization among Chinese children. Given the aforementioned limitations, we agree that further investigations on the molecular mechanisms linking mitochondrial respiratory chain enzymes and pediatric sepsis are warranted.

## Conclusion

Low serum CoQ10 and decreased mitochondrial complex I + III activity can predict the sepsis incidence and related mortality among Chinese children.

## Supplementary Information

**Additional file 1: eFile S1.** The STROBE checklist.

**Additional file 2: eFile S2.** Definitions of systemic inflammatory response syndrome (SIRS), infection, sepsis.

## Data Availability

The datasets used and/or analysed during the current study are available from the corresponding author on reasonable request.

## Abbreviations

CoQ10: Coenzyme Q10
PICU: Pediatric Intensive Care Unit
SOFA: Sequential organ failure score
PCIS: Pediatric critical illness score
ARDS: Acute respiratory distress syndrome
LD: Lactate dehydrogenase
HBDH: Hydroxybutyrate dehydrogenase
AIC: Akaike information criterion
BIC: Bayesian information criterion
NRI: Net reclassification improvement
IDI: Integrated discrimination improvement
ROC: Receiver operating characteristic
OR: Odds ratio
CI: Confidence interval
*I*^2^: Inconsistency index
