# Supplementary material for: Association of mitochondrial respiratory chain enzymes with the risk and mortality of sepsis among Chinese children
Source: BMC Infect Dis. 2022 Jan 6;22:34. doi: 10.1186/s12879-021-07014-6 (PMC8740061; doi:10.1186/s12879-021-07014-6)
Supplement: Supplementary file 2 — Additional file 2: eFile S2. Definitions of systemic inflammatory response syndrome (SIRS), infection, sepsis. [file 12879_2021_7014_MOESM2_ESM.docx]

**eFile 2.** Definitions of systemic inflammatory response syndrome (SIRS), infection, sepsis.

| SIRS  The presence of at least two of the following four criteria, one of which must be abnormal temperature or leukocyte count:  ● Core temperature of 38.5℃ or 36℃.  ● Tachycardia, defined as a mean heart rate＞2SD above normal for age in the absence of external stimulus, chronic drugs, or painful stimuli; or otherwise unexplained persistent elevation over a 0.5- to 4-hr time period OR for children <1yr old: bradycardia, defined as a mean heart rate  <10th percentile for age in the absence of external vagal stimulus,β-blocker drugs, or congenital heart disease; or otherwise unexplained persistent depression over a 0.5-hr time period.  ● Mean respiratory rate ＞2SD above normal for age or mechanical ventilation for an acute process not related to underlying neuromuscular disease or the receipt of general anesthesia.  ● Leukocyte count elevated or depressed for age (not secondary to chemotherapy-induced leukopenia) or ＞10% immature neutrophils.  Infection  A suspected or proven (by positive culture, tissue stain, or polymerase chain reaction test) infection caused by any pathogen OR a clinical syndrome associated with a high probability of infection. Evidence of infection includes positive findings on clinical exam, imaging, or laboratory tests (e.g.）,white blood cells in a normally sterile body fluid, perforated viscus, chest radiograph consistent with pneumonia, petechial or purpuric rash, or purpura fulminans)  Sepsis  SIRS in the presence of or as a result of suspected or proven infection. |
| --- |
